# Hyperspectral Imaging Using Flexible Endoscopy for Laryngeal Cancer Detection

**DOI:** 10.3390/s16081288

**Published:** 2016-08-13

**Authors:** Bianca Regeling, Boris Thies, Andreas O. H. Gerstner, Stephan Westermann, Nina A. Müller, Jörg Bendix, Wiebke Laffers

**Affiliations:** 1 Laboratory for Climatology and Remote Sensing, Faculty of Geography, University of Marburg, Deutschhausstr. 12, Marburg 35032, Germany; thies@staff.uni-marburg.de (B.T.); bendix@staff.uni-marburg.de (J.B.); 2 Klinikum Braunschweig, ENT-Clinic, Holwedestr. 16, Braunschweig 38118, Germany; gerstaoh@web.de; 3 Department of Otorhinolaryngology/Head and Neck Surgery, University of Bonn, Sigmund-Freud-Str. 25, Bonn 53127, Germany; stephan.westermann@ukb.uni-bonn.de (S.W.); nina-alexa.mueller@ukb.uni-bonn.de (N.A.M.); wiebke.laffers@gmx.de (W.L.)

**Keywords:** flexible endoscopy, honeycomb-like pattern removal, laryngeal cancer detection, classification

## Abstract

Hyperspectral imaging (HSI) is increasingly gaining acceptance in the medical field. Up until now, HSI has been used in conjunction with rigid endoscopy to detect cancer in vivo. The logical next step is to pair HSI with flexible endoscopy, since it improves access to hard-to-reach areas. While the flexible endoscope’s fiber optic cables provide the advantage of flexibility, they also introduce an interfering honeycomb-like pattern onto images. Due to the substantial impact this pattern has on locating cancerous tissue, it must be removed before the HS data can be further processed. Thereby, the loss of information is to minimize avoiding the suppression of small-area variations of pixel values. We have developed a system that uses flexible endoscopy to record HS cubes of the larynx and designed a special filtering technique to remove the honeycomb-like pattern with minimal loss of information. We have confirmed its feasibility by comparing it to conventional filtering techniques using an objective metric and by applying unsupervised and supervised classifications to raw and pre-processed HS cubes. Compared to conventional techniques, our method successfully removes the honeycomb-like pattern and considerably improves classification performance, while preserving image details.

## 1. Introduction

Squamous cell carcinoma is the most common head and neck cancer and the cause of approximately 350,000 deaths per annum worldwide [1]. Early detection of the tumor greatly increases the chance of a successful treatment. Conventional white light endoscopy often leads to misdetection of malign lesions at an early stage. As a result, a great deal of effort has been invested into developing new examination methods, such as narrow band imaging (NBI) [2,3]. NBI combines two specific wavelengths into a single pseudo-color image to visualize the differences in epithelium quality and changes in mucosal vascularization. Similar to NBI, hyperspectral imaging (HSI) could be the next powerful optical technique in cancer detection. HSI utilizes a wider range of the electromagnetic spectrum than NBI, providing high-resolution spectral and spatial information. A stack of images depicting the same area over a specified interval of wavelengths forms the so-called hyperspectral (HS) cube. Its pixels provide the spectral signature of the underlying tissue. Since disease-related alterations in tissue physiology and morphology affect the tissue’s reflectance properties, it is possible to identify and classify different biological tissues through their characteristic signatures. HSI aids in gathering this spectral information that is relevant to diagnoses around a wide area of the underlying tissue. HSI is gradually gaining wider acceptance in the field of medicine. Various studies have already exhibited its value, for example, the characterization of burns [4], estimating skin thickness [5], intraoperative visualization of cerebral oxygenation [6] and diabetic foot ulcers [7]. In cancerous tissue detection, HSI has already been applied both in and ex vivo [8,9,10,11,12,13,14]. However, previously published studies have only used an HS system in combination with a rigid endoscope. As a logical next step, HSI should be paired with flexible endoscopy to improve access to hard-to-reach areas. Pairing the HS system with the flexible endoscope presents a unique combination of optical devices that has not previously been used. A flexible endoscope consists of thousands of fiber optic cables, which provide the advantage of flexibility but simultaneously introduce an interfering honeycomb-like pattern onto the images. Due to the strong impact that these patterns have on classification performance and other image processing methods, it is imperative to correct them before digital image classification approaches can properly detect cancer. To identify even small variations and changes of the tissue, high image resolution is necessary. Therefore, it is crucial that the honeycomb-like pattern must be removed accurately, but image details must be preserved.

Previous studies on honeycomb-like pattern removal from endoscopic images have either filtered in the spatial [15] or Fourier domain. Spatial domain (SD) filtering reduces the image resolution, since only low frequencies are passed, while high frequencies are removed. For Fourier domain (FD) filtering, [16] designed circular and star-shaped masks that let only low frequencies pass, but which also clearly reduce the resolution of an image. Other studies have exploited the fact that, in the FD, periodic and quasi-periodic noise appear as regularly distributed areas of high amplitudes with a single peak in its center. Thus, only small local image areas are affected by the noise as opposed to the SD, where the whole image is affected. When the noise-affected frequencies are filtered exclusively, most high and low frequencies remain unaffected, which reduces information loss. [17] performed an auto-search algorithm to identify the peaks and designed a mask of notch-reject filters of pre-defined sizes; and [18] designed windowed Gaussian filters of pre-defined sizes centered at the peaks. Both studies do not take into account the varying spatial extension of the affected components. However, in order to sufficiently remove the honeycomb-like pattern while simultaneously minimizing the loss of information in an image, the size of the filter must be individually determined for each peak. The main goal of this study is to introduce a filter technique that minimizes the loss of information as much as possible by simultaneously suppressing the honeycomb artifact in HS images for an improvement of laryngeal cancer detection. For this purpose, we designed a method to detect the peaks and identify the spatial extent of the noise-affected frequencies to determine the optimal filter size. We tested various designs, such as Gaussian and ideal, to find the most appropriate filter. We compare our method of pattern removal to the widely used method of Gaussian filtering in the SD and star-shaped filtering proposed by [16] using HS data from a United States Air Force (USAF) test chart. Thereafter, we applied unsupervised and supervised classifications with a Gaussian mixture model (GMM) and similarity measure to laryngeal HS cubes to document the impact of the new pre-processing procedure on the hyperspectral classification results.

In addition to the honeycomb-like pattern, HS data collected with a flexible endoscope faces the same problems as HS data recorded by rigid endoscopy. These issues include false registration from one image to the next within the HS cube due to the patient’s heartbeat, white noise in the lower wavelength spectrum and specular reflections (SR). Image contaminations can, however, be successfully corrected by a pre-processor that was recently developed [19]. The honeycomb-like pattern removal technique is included in the pre-processor to make it applicable to HS imaging with a flexible endoscope.

This paper is organized as follows: Section 2 outlines the details of the instrumentation and patients. Section 3 describes the new filtering method and, in addition, outlines further pre-processing steps and the classification settings. Section 4 describes the results of the pattern removal method as well as of the classification. Our conclusion is then presented in the final section.

## 2. Instrumentation and Patients

**Figure 1:**
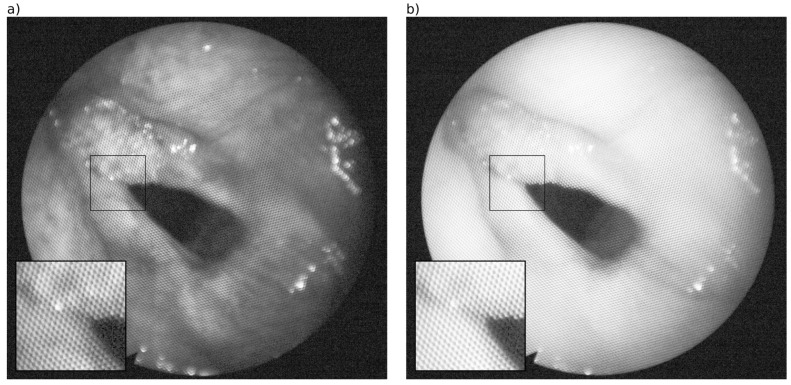
Hyperspectral (HS) images of the larynx of one example case (#caseA) at wavelength (**a**) 510 nm and (**b**) 610 nm showing the honeycomb-like pattern as a result of the hexagonally arranged fiber cables in the flexible endoscope.

For hyperspectral imaging, a Polychrome V monochromator (TILL Photonics GmbH, Graefelfing, Germany) was triggered synchronously with a monochromatic high-resolution CCD-camera (Axiocam MRm, Carl Zeiss MicroImaging GmbH, Oberkochen, Germany). A flexible endoscope was attached to the monochromator and the camera using a fiber optic light cable. Both devices were controlled using commercially available software (AxioVision 4.8, Carl Zeiss MicroImaging GmbH). To reduce movement artifacts, the endoscope was held in place by an endoscope holding system. The system generates HS cubes between 390 and 680 nm in 30 iterative steps with a 10 nm interval. HS images measure 1388 × 1040 pixels with a bit depth of 12. The flexible endoscope is made of thousands of hexagonally arranged fibers. Each optical fiber consists of a core and a cladding, which have a higher and a lower refractive index, respectively, and generates total reflection at the core-cladding interface. Light is transmitted from where it enters the fiber to the other end by following the bends through the core without a loss in intensity. The image shows honeycomb-like structures, consisting of bright, circular-shaped patterns surrounded by dark rings analogous to the arrangement of the fibers and the relation of the core and cladding (Figure 1). Patients undergoing routine endoscopy for diagnostic purposes were imaged using the method described above. All experimental procedures were approved by the Ethical Committee of the Rheinische Friedrich–Wilhelms-University Bonn and informed consent was obtained from every participating patient. This study considers two HS cubes (hereinafter referred to as #caseA and #caseB) from patients with histopathological diagnoses of head and neck squamous cell carcinoma.

## 3. Methods

This section provides a detailed description of the method for removing the honeycomb-like pattern as well as additional pre-processing steps and the classification methods. Removing the honeycomb-like pattern prior to processing the image further (such as image registration) is important, since the pattern is superimposed over the other data structures. For additional image improvement, the pre-processor developed by [19] was applied to the HS data to correct the other data interferences mentioned in the introduction. In addition, we applied a normalized ratio index (NRI) to effectively reduce the impact of non-uniform illumination conditions in the HS cubes. Finally, unsupervised and supervised classifications were applied to the exemplary cases.

### 3.1. Removal of Honeycomb-Like Pattern in the Fourier Domain

Periodic and quasi-periodic noise appear as concentric bursts of energy at the position corresponding to the frequencies of periodic interference in the FD [20]. Frequencies corresponding to noise are recognizable by higher amplitudes than surrounding components and a high center point, the so-called peak. Filtering the noise-affected frequencies removes the periodic and quasi-periodic noise in the SD.

**Figure 2:**
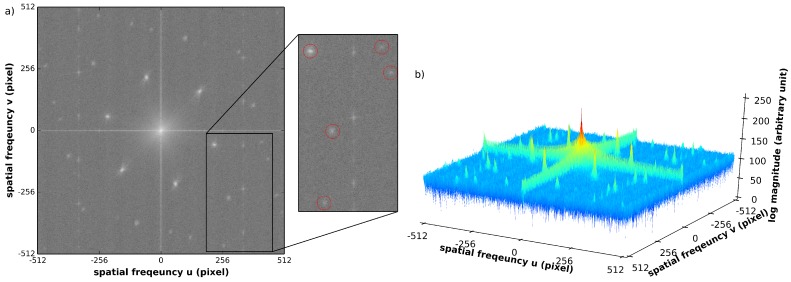
(**a**) Fourier spectrum of the HS image at wavelength 510 nm corresponding to Figure 1a. The affected components are marked by red circles for the zoomed area; and (**b**) Fourier spectrum presented as a 3D graphic.

The honeycomb-like pattern produces six radial, symmetrically arranged and clearly visible peaks in the lower frequencies around the direct current (DC) (Figure 2). Depending on the individual image characteristics, less visible and more difficult to detect peaks are located in the higher frequencies. The noise-affected frequency components vary in location, shape and extension within and between the HS cubes. However, they must be detected and removed to successfully eliminate the honeycomb-like pattern. To avoid an unnecessary loss of information due to filtering, selective filters were specifically designed for each peak. According to the approach described by [18], filtering is done in windows of size n×n pixels, of which the peak is the central point. To adjust the filter to the size of the affected components, information about their location and extension is needed. In order to consider these points, we developed a method for selective filtering with an optimal filter size. The proposed method includes the following steps: (i) identification of the peaks; (ii) identification of affected components to derive filter size and (iii) filtering in the FD.

**Figure 3:**
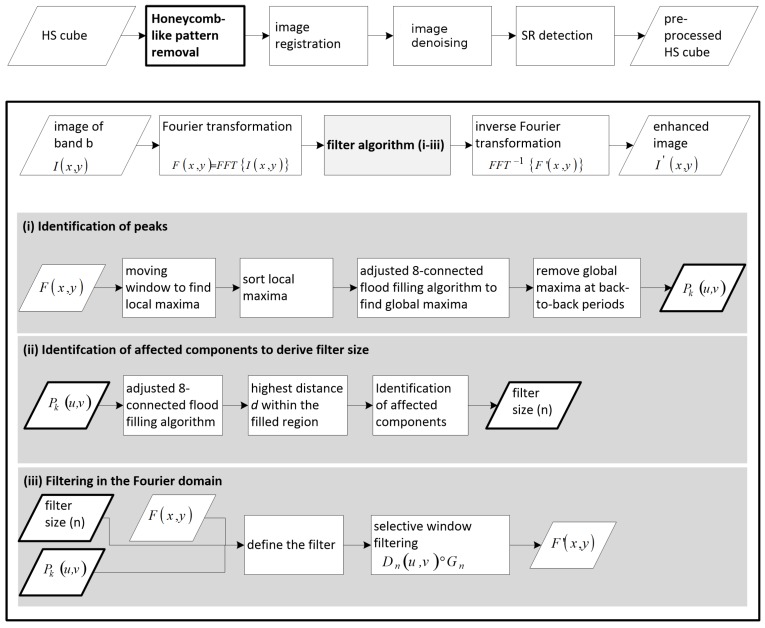
Flow chart of the proposed method for honeycomb-like pattern removal.

The FD image was computed from the original SD image using a 2D fast Fourier transform (FFT) algorithm. The procedure is explained in detail in the following sections and depicted in Figure 3.

#### 3.1.1. Identification of Peaks

The challenging task of peak detection was solved in the following manner. Local maxima were identified on the Fourier spectrum by comparing each pixel to its neighboring pixels using a moving window of 3 × 3 pixels. If a pixel has a higher value than its neighbors, it is defined as a local maximum. To derive peaks from the local maxima, the latter were used as seed points for an eight-connected flood-fill algorithm. Starting with the local maximum of the highest amplitude, flood filling was successively applied to each local maximum in descending order of amplitude. A pixel was included in the flooded region if its value was less than or equal to the value of the local maximum and greater than or equal to the local maximum minus an empirically determined value *t*. A local maximum was not considered as a global maximum if a pixel was reached that was already filled during flooding of another local maximum. Another exclusion criterion for a particular local maximum was the hit of a pixel with an amplitude higher than that local maximum. This approach adapts the global maxima detection algorithm of the open source software ImageJ (1.49) [21]. The resulting peak list was cleaned due to falsely identified peaks at back-to-back periods, where the amplitude is relatively high compared to the overall Fourier spectrum. Filtering the corresponding components would have led to image blurring, so it was important to exclude them from further steps. The result is a list of *k* peaks.

#### 3.1.2. Identification of Affected Components to Derive Filter Size

To derive an individual size *n* for each filter window, an eight-connected flood-fill algorithm was adapted to identify the extent of the affected components corresponding to each peak. The algorithm incorporates new pixels into a region if their value is less than the value of the current pixel. Peaks were used as starting points for flood filling and the resulting connected region marks the affected neighborhood. Next, the highest distance *d* between the peak and a pixel within the flooded region was identified. This distance was used to define the area $D_{n}^{uv}$ of size *n* around the peak $P_{k}^{uv}$ that should be filtered. The size of the n×n pixel window is defined by n=2da+1, where *a* is an experimentally determined scaling factor.

#### 3.1.3. Filtering in the Fourier Domain

Affected components were filtered using the windowed filtering approach [18]. The frequency area $D_{n}^{uv}$ around $P_{k}^{uv}$ is filtered by a windowed filter as follows:

(1) $${\tilde{D}}_{n}^{uv}=D_{n}^{uv}\circ G_{n},$$

wherein ${\tilde{D}}_{n}^{uv}$ is the corresponding frequency area after filtering and $G_{n}$ is the filter matrix of n×n pixels.

**Figure 4:**
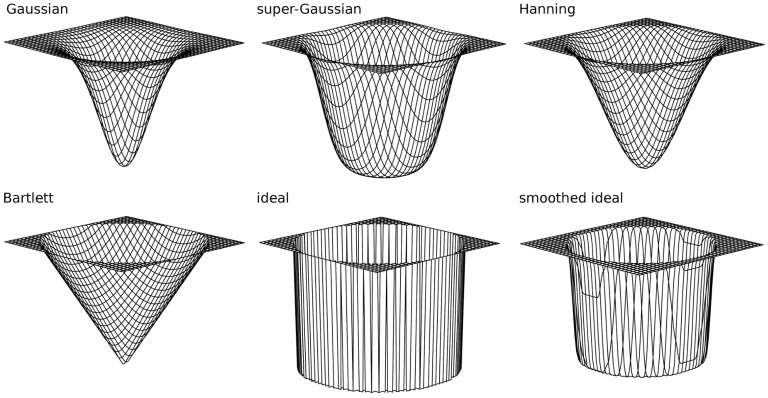
Various filters used for performance test.

We tested several different filtering techniques (Figure 4), including Gaussian (2), super-Gaussian (3), Hanning (4), Bartlett (5), ideal (6) and smoothed ideal [22]:

(2) $$G_{n}\left(x,y\right)=1-e^{\frac{-r_{xy}^{2}}{2\sigma^{2}}},\;\sigma =0.3,$$

(3) $$G_{n}\left(x,y\right)=1-e^{\frac{-r_{xy}^{b}}{\kappa }},\;\kappa =0.3,b=6.0,$$

(4) $$G_{n}\left(x,y\right)=\left\{\begin{array}{cc} 1-0.5[cos\left(\pi r_{xy}\right)+1] & \;\text{if}\;0\leq r_{xy}\leq 1,\\ 1 & \;\text{else} \end{array}\right.$$

(5) $$G_{n}\left(x,y\right)=\left\{\begin{array}{cc} r_{xy} & \;\text{if}\;0\leq r_{xy}\leq 1,\\ 1 & \;\text{else} \end{array}\right.$$

(6) $$G_{n}\left(x,y\right)=\left\{\begin{array}{cc} 0 & \;\text{if}\;0\leq r_{xy}\leq 1,\\ 1 & \;\text{else} \end{array}\right.$$

where *x* and *y* are the coordinates within the filter window and $r_{xy}=\sqrt{{\left(\frac{x-(n-1)/2}{(n-1)/2}\right)}^{2}+{\left(\frac{y-(n-1)/2}{(n-1)/2}\right)}^{2}}$. We compared these filter designs in order to identify the most appropriate approach for removing the honeycomb-like pattern while preserving image details. The smoothed ideal filter was computed by the convolution of the ideal filter using a Gaussian kernel of 9 × 9 pixels. In addition, our method was compared to Gaussian filtering with various kernel sizes in the SD and Gaussian smoothed star-shaped filtering as described by [16]. This is an ideal low-pass filter in the form of a star, with convex corners located between the six radial symmetric peaks around the DC and smoothed by Gaussian filtering (with a kernel size of 9 × 9 pixels).

To quantify the performance of the filter techniques, quality metrics were computed on the basis of the USAF test chart. This is a resolution test pattern that consists of several groups of different-sized bars and was originally developed by the USAF to test the resolution of optical imaging systems. The HS cube of the USAF test chart was recorded under exclusion of external light sources to emulate the light conditions of the larynx. The comparison was made using the quality metrics proposed by [16], variance-based smoothness *s* and Rayleigh-based line separation criteria *r*. The combination of both is given by the weighted quality measure q=γ∗s+(1−γ)∗r, where *γ* is the factor that weights the importance of *s* compared to *r*. The quality metrics ranges between 0 and 1. As the value of *q* increases, the better the performance is in terms of *γ*. The metric takes into account both the positive effects of removing the honeycomb-like pattern as well as the negative impact of blurring. *s* measures how well the honeycomb-like pattern is removed by comparing the average standard deviation of a homogeneous area of the filtered image to the unfiltered image, while *r* measures the detail-preserving quality of the filter, which can be derived from the filtered image of a USAF test chart using an image area along line patterns. The less those line patterns are blurred, the higher the detail resolution of the filtered image is.

### 3.2. Further Pre-Processing and Hyperspectral Classification

#### 3.2.1. Application of the Image Pre-Processor

In addition to the honeycomb-like pattern, the HS data exhibit other complications, which were corrected by the pre-processor proposed by [19]. The pre-processor was designed for HS recordings made by a rigid endoscope. We adapted the individual modules of the pre-processor, image registration, image denoising and detection of SR, for flexible endoscopy. Image registration was used to reduce the shift of individual images within the HS cube caused by sequential scanning with the camera while the patients’ tissue moves due to their heartbeat. Image denoising was applied to reduce the white noise in the lower wavelengths. The specular reflection method identifies and removes image areas with SR from the HS data to avoid impacting the classification. However, the combination of the honeycomb-like pattern and the strong white noise in the lower wavelengths reduces the signal-to-noise ratio in the first two bands to an extent that made satisfactory correction impossible. Hence, these two bands were excluded from further processing steps.

#### 3.2.2. Illumination

The combination of the anatomy of the larynx and the geometry of light source results in various intensities of light reaching the mucosa surface. If a spectrally homogeneous object is variably illuminated, its spectral signatures are linearly scaled variations of one another. Although the shape of the spectral signature remains the same regardless of the illumination conditions, there is a significant impact on classification performance. To effectively reduce image artifacts caused by non-uniform illumination of the scene, NRI [23] Equation (7) was calculated from all possible two-band combinations of HS cubes between 410 and 680 nm:

(7) $$nri_{\left(\lambda 1,\lambda 2\right)}=\frac{I_{\lambda 1}-I_{\lambda 2}}{I_{\lambda 1}+I_{\lambda 2}},\;\lambda 1>\lambda 2,$$

where λ1 and λ2 are spectral bands of the HS cube.

#### 3.2.3. Hyperspectral Classification

Unsupervised classification with GMM and supervised classification with spectral similarity measure were applied to the raw and pre-processed HS data to test the performance of the introduced filter technique in terms of HS classification. We compared our method with the super-Gaussian filter, SD-Gaussian filter with a kernel size of 17 × 17 pixels and star-shaped filter.

The GMM is an established unsupervised classification method in HS imaging, wherein each cluster corresponds to a Gaussian distribution. The GMM is a parametric probability density function defined by

(8) $$p\left(x\right)=\sum_{k=1}^{k}\alpha_{k}N\left(X,\mu_{k},\sum_{k}\right),$$

wherein *X* denotes the feature vector and *k* is the number of mixture components, namely, the number of clusters. $\alpha_{k}$,$\mu_{k}$,$\sum_{k}$ are the mixing weights, mean vector and covariance matrix of the *k*th component, which is expressed by $N(X,\mu_{k},\sum_{k})$. The maximum likelihood estimates of these unknown parameters were found by the expectation-maximization (EM) algorithm proposed by [24]. EM iterations stop once a pre-defined convergence threshold is reached. GMM with full covariance matrices was fitted on the NRI spectra extracted from the raw and pre-processed HS cube. SR were detected from the pre-processed HS cube and excluded from the unsupervised classification. GMM was performed for 10 clusters.

Supervised classification was used to visualize the effect of filtering on the information content of HS images. Therefore, a spectral similarity measure was used to detect the cancerous tissue of #caseA using the mean NRI spectrum of the cancerous tissue of the second case (#caseB). The mean NRI spectrum was calculated from 100 randomly selected pixels within the marked area of cancerous tissue. We used the Pearson’s correlation coefficient as a similarity measure, since it is responsive to the spectral shape but not to changes in brightness. The correlation coefficient ranges between −1 and 1, negative and positive correlation, respectively; 0 indicates no relationship between spectra.

## 4. Results and Discussion

The proposed method for removal of the honeycomb-like pattern was tested for various filters and compared to other methods. The USAF test chart was used to evaluate the method’s efficacy in removing the desired pattern while simultaneously preserving image details and compute quality metrics. Table 1 lists the two measures *s* and *r*, as well as the resulting combined measure *q* for different weights *γ*, which are 0.5 and 0.8. In the case of γ=0.5, the weight is equally balanced between detail-preserving and smoothness, whereas γ=0.8 shifts more weight to the smoothness. Table 1 shows the mean and standard deviation of the quality metrics for the entire HS cube. Removing the pattern from the USAF test chart through filtering leads to image smoothing that corresponds to a higher *s* value. If no filter is applied, the image reaches its highest value for detail preservation *r*, since smoothing does not occur. *s* is 0 for the unfiltered image, since it was used as reference.

**Figure 5:**
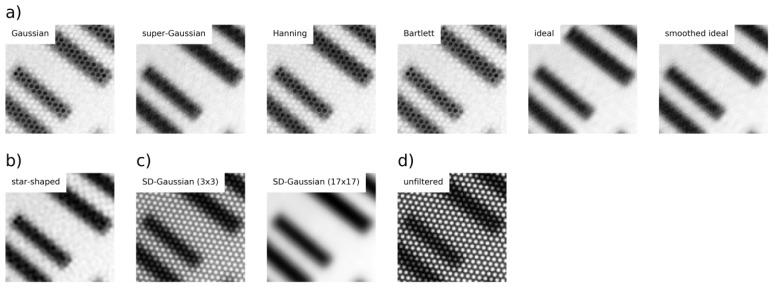
Test chart displayed in detail at wavelength 510 nm for (**a**) our method using different filters; (**b**) star-shaped filtering (**c**) filtering in the SD using Gaussian filtering with two different kernel sizes and (**d**) the unfiltered image.

The evaluation reveals that our method, in combination with the various filters described above, removes the pattern by different degrees. In detail, super-Gaussian filtering delivers the best results for pattern removal (s=0.858), while Gaussian filtering best preserves the details (r=0.638). Both filters return equal results for γ=0.5, while super-Gaussian filtering outperforms all other filters at γ=0.8. The visual inspection (Figure 5) underlines the failure of the Gaussian and Bartlett filters in removing the pattern in highly contrasted areas, resulting in a low *s*. However, super-Gaussian filtering achieves good results in smoothing as well as detail preservation. The small standard deviation of *q* demonstrates that there are only slight differences in quality among the images of the same HS cube. Similar to the super-Gaussian filter, Hanning, ideal and smoothed ideal filtering achieve high values for *s*, but low values for *r*. In addition, ideal and smoothed ideal filtering leads to ring artifacts on the edges of the image.

Star-shaped filtering obtains smaller values for γ=0.5 and for γ=0.8 than our proposed method. However, visual inspection reveals that this approach does not completely remove the honeycomb-like artifact.

SD-Gaussian filtering is only able to remove the pattern using large filter kernels. For the USAF test chart, the kernel size requires a minimum of 17 pixels to satisfactorily remove the pattern, but it also leads to higher detail preservation as shown in Figure 5c. The strong image blurring effect is reflected by high values for *s*, and a simultaneously considerable low value for *r*. In contrast, a filter kernel of 3 × 3 pixels does not remove the pattern. In summary, SD-Gaussian filtering either leads to strong image blurring or an unsatisfactory removal of the pattern.

Overall, the quality metrics signify that our method, especially in combination with the super-Gaussian filter, is more efficacious in suppressing the pattern than Gaussian filtering in the SD and star-shaped filtering in the FD.

**Figure 6:**
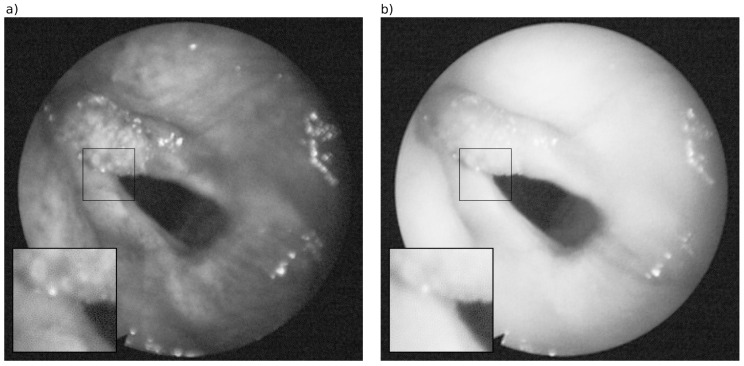
HS images of the larynx (#caseA) after removing the honeycomb-like pattern by our method at wavelength (**a**) 510 nm and (**b**) 610 nm (corresponding to the images in Figure 1).

**Figure 7:**
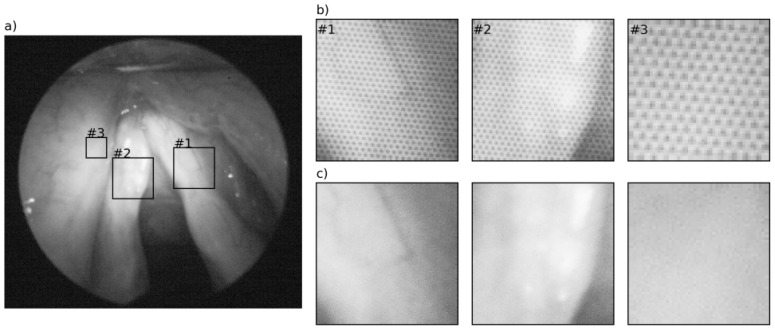
(**a**) HS image of the larynx (#caseB) at wavelength 510 nm after removal of the pattern, zoom in areas (**b**) before and (**c**) after removal of the pattern.

Applying our method to the HS cubes of the larynx likewise results in good image quality, as a visual examination of the two example cases reveals. Figure 6 depicts the HS images of two wavelengths after pattern removal using our method with the super-Gaussian filter. A comparison with the corresponding HS images before pattern removal (see Figure 1) demonstrates that the pattern is completely removed, while the image details are preserved. The same applies to the second exemplary case (Figure 7), where the quality of the image was distinctly improved.

**Figure 8:**
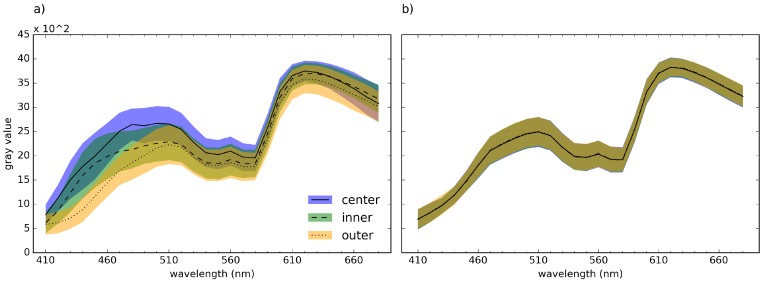
Mean and standard deviation of spectra from healthy tissue extracted from the center, the inner and the outer pixels of 800 honeycombs (**a**) before and (**b**) after pattern removal.

To analyze how the filtering affects the pixel spectra, we extracted the spectra of 800 honeycombs in the unfiltered and filtered HS cubes of healthy tissue in #caseA. The spectra were extracted from the center, the inner and the outer pixel of the honeycombs. Figure 8 shows the mean and standard deviation of the spectra of the pixel corresponding to the honeycombs before (Figure 8a) and after pattern removal (Figure 8b). Figure 8a underlines that the spectra of the center, the inner and the outer pixels vary in brightness and shape. The brightness decreases from the center to the outer pixel in most wavelengths. Examining the spectra after the removal of the honeycomb-like pattern in Figure 8b, it becomes clear that the filtering leads to a change in shape and brightness of the spectra, whereby the spectra of the inner and the outer pixels increase in brightness while the center pixels become darker.

**Figure 9:**
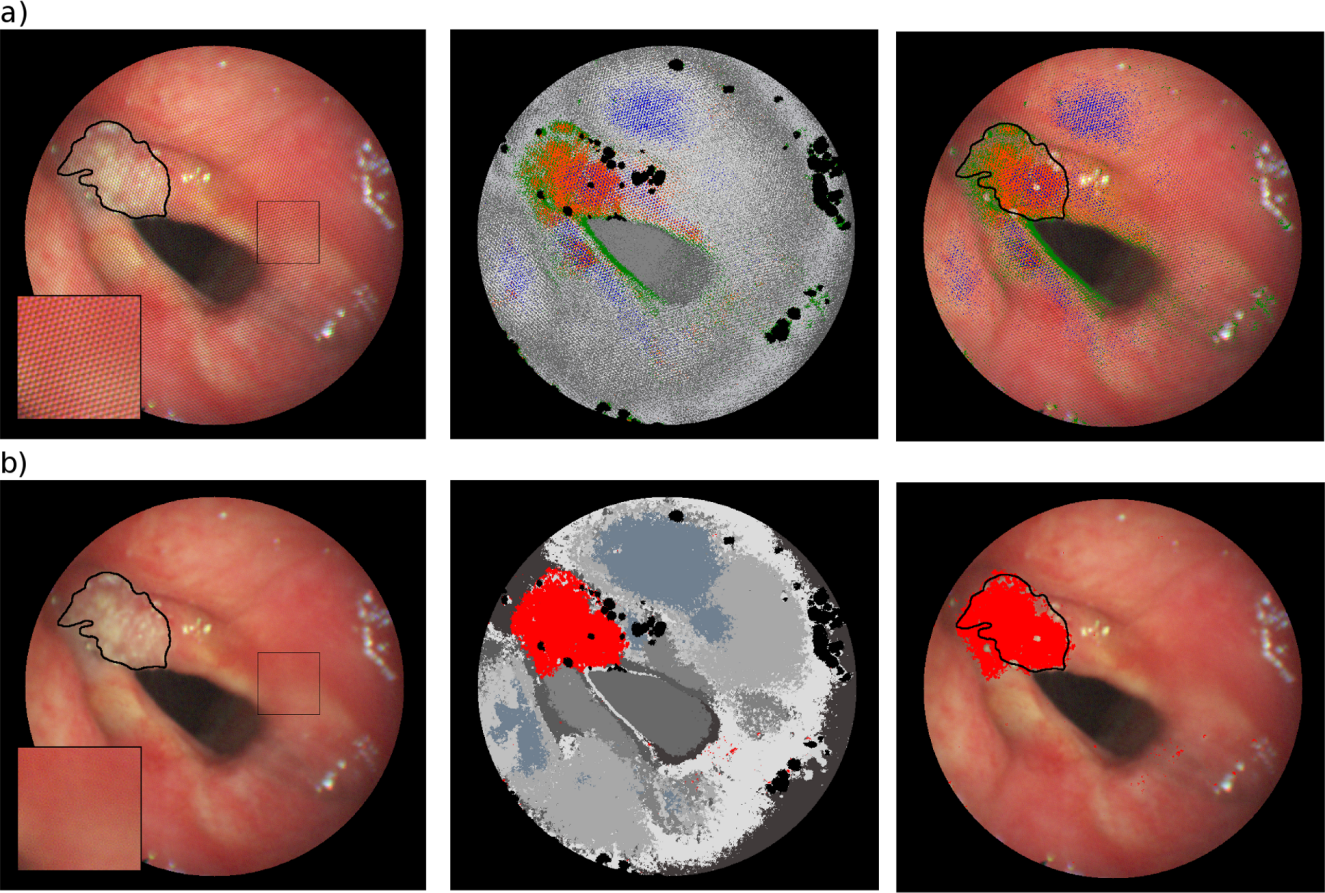
Unsupervised classification results for #caseA (**a**) before and (**b**) after pre-processing. Red Green Blue (RGB) image of the HS cube with cancerous tissue marked by a black line (left), unsupervised classification results with the cluster/clusters corresponding to a cancerous area marked in color (middle) and overlay of the cluster/clusters corresponding to the cancerous area and the RGB image (right).

Unsupervised classification by GMM was applied to the NRI of the raw and the pre-processed HS cube of one example case (#caseA). Figure 9 presents the classification results. Clusters corresponding mainly to the cancerous area are displayed in color. In comparing the classification results before and after pre-processing, it becomes clear that the pattern has a significant impact on classification performance. As Figure 9a illustrates, the data’s separation into clusters not only depends on the signature of the tissue, but also on the honeycomb-like pattern, which is clearly reflected in the classification results, since the clusters seem more dependent on the pattern than on the underlying signature. The clusters corresponding to the marked region clearly overestimate the cancerous tissue. For example, the class displayed in blue includes pixels from the cancerous tissue as well from the cancer-free vestibular folds. The pre-processed HS data is illustrated in Figure 9b, where the cancer is clearly outlined and incorrect classifications are reduced.

**Figure 10:**
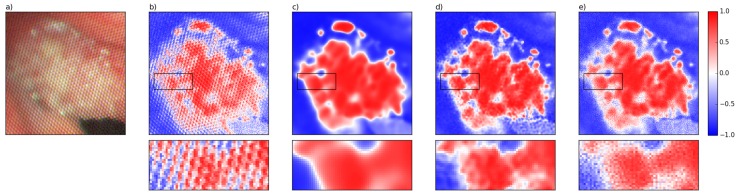
Results of cancerous tissue detection by hyperspectral classification using correlation for the zoomed area which shows the cancerous tissue of #caseA (**a**). As underlying data for classification, we used (**b**) raw data; (**c**) SD Gaussian filtered data; (**d**) star-shaped filtered data; and (**e**) data filtered by our method.

Figure 10 displays the results of the supervised classification of #caseA using raw data, SD-Gaussian filtered data, star-shaped filtered data and data filtered by our method. The closer the correlation coefficient is to one, the higher the pixel is correlated to the cancerous tissue of #caseB. A correlation coefficient approaching −1 indicates a negative correlation to the cancerous tissue of #caseB. For classification of the raw data (Figure 10b), the pattern is clearly reflected in classification results, in particular on the margins of the cancerous tissue. SD-Gaussian filtering (Figure 10c) removes the honeycomb-like pattern, but greatly blurs the details since the filter only passes low frequencies. Structures of small spatial expansions are either blurred or entirely eliminated. Star-shaped filtering (Figure 10d) returns better results in terms of blurring and preservation of information than SD-Gaussian filtering, since it passes more frequencies, but the classification results illustrate that blurring and the honeycomb-like pattern are still present. Our filtering technique (Figure 10e) achieved the best results. The details of the image are highly preserved and the honeycomb-like pattern is completely removed. Ultimately, this leads to a highly differentiated classification result.

## 5. Conclusions

This study is the first of its kind to attach an HS system to a flexible endoscope to detect laryngeal cancer. As a prerequisite for proper detection, we proposed a pre-processing method to improve the HS data for further analysis. This feasibility study was conducted with the overall objective of creating a spectral distinction between cancerous and non-cancerous tissue in HS data by utilizing the advantages of the flexible endoscope. The major disadvantage to this system, however, is that honeycomb-like patterns appear and degrade the quality of the HS images. This can be attributed to the fiber optic cables, which make the endoscope flexible yet prevent successful classification without a correction of the images’ degradation. Therefore, we developed a method specifically for the system in order to overcome this drawback, in which the spectral domain is filtered by a selective window that automatically adjusts to the size of the periodic noise. Our method clearly shows better results in terms of pattern smoothing and preserving detail compared with other methods. Using the pre-processed HS data as an input for an unsupervised and supervised classification instead of the raw HS cubes improves classification performance and the ability to locate the cancerous tissue, as revealed by the visual inspection. However, clinical studies are necessary to optimize cancer detection.

## Figures and Tables

**Table 1:** Comparison of our method using various filters to spatial domain (SD)-Gaussian filtering and star-shaped filtering to remove the honeycomb-like pattern from the United States Air Force (USAF) test chart. Mean and standard deviation are listed for quality metrics Rayleigh-based line separation criteria (*r*), variance-based smoothness (*s*) and quality measure (*q*) of the HS cube.

	*s*	*r*	*q* (γ=0.5)	*q* (γ=0.8)
**Our Method**				
Gaussian	0.820 (0.054)	0.638 (0.066)	0.729 (0.030)	0.784 (0.039)
Super-Gaussian	0.858 (0.056)	0.600 (0.044)	0.729 (0.028)	0.806 (0.042)
Hanning	0.851 (0.059)	0.572 (0.105)	0.711 (0.067)	0.796 (0.057)
Bartlett	0.827 (0.053)	0.617 (0.059)	0.722 (0.028)	0.785 (0.038)
Ideal	0.851 (0.060)	0.572 (0.105)	0.711 (0.067)	0.796 (0.057)
Smoothed ideal	0.856 (0.056)	0.586 (0.071)	0.721 (0.044)	0.802 (0.046)
**Star-shaped**	0.795 (0.044)	0.622 (0.065)	0.709 (0.024)	0.760 (0.028)
**SD-Gaussian**				
kernel size: 3 × 3	0.692 (0.006)	0.672 (0.036)	0.682 (0.018)	0.688 (0.008)
kernel size: 17 × 17	0.936 (0.025)	0.408 (0.041)	0.672 (0.019)	0.831 (0.018)
**Unfiltered**	0.000 (0.000)	0.736 (0.030)	0.368 (0.015)	0.147 (0.006)

## Abbreviations

The following abbreviations are used in this manuscript:

DC	direct current
EM	expectation-maximization
FD	Fourier domain
FFT	fast Fourier transform
GMM	Gaussian mixture model
HS	hyperspectral
HSI	hyperspectral imaging
NBI	narrow band imaging
NRI	normalized ratio index
RGB	Red Green Blue
SD	spatial domain
SR	specular reflection
USAF	United States Air Force
